# Cystatin C: A Strong Marker for Lower Limb Ischemia in Chinese Type 2 Diabetic Patients?

**DOI:** 10.1371/journal.pone.0066907

**Published:** 2013-07-02

**Authors:** Fang Liu, Jing Shen, Jun Zhao, Hui Zeng, Lianxi Li, Jungong Zhao, Fengdi Lu, Yuqian Bao, Weiping Jia

**Affiliations:** Department of Endocrinology & Metabolism, Shanghai Jiaotong University Affiliated Sixth People’s Hospital, Shanghai Clinical Center for Diabetes, Shanghai Diabetes Institute, Shanghai Key Laboratory of Diabetes Mellitus, Shanghai Key Clinical Center for Metabolic Diseases, Shanghai, China; University of Hong Kong, China

## Abstract

**Objective:**

Cystatin C is growing to be an ideal indicator for renal function and cardiovascular events. The aim of this study was to investigate the relationship between serum cystatin C levels and peripheral arterial disease and to explore its diagnostic value for lower limb ischemia (LLI) in type 2 diabetic population.

**Methods:**

A total of 1609 T2DM patients were included in this cross-sectional study. Their clinical and biochemical characteristics, ankle-brachial index (ABI), carotid and lower extremity arterial ultrasound were detected. LLI was defined by ABI <0.9 and lower extremity arterial stenosis >50% by ultrasound examination. Patients were divided to two groups: with LLI and without. The risk factors of LLI were explored by binary logistic regression analysis.

**Results:**

The serum concentrations of cystatin C were 1.53±0.60 and 1.08±0.30 mg/L in patients with and without LLI, respectively. Binary logistic regression analysis showed that the significant risk factors were cystatin C *(P* = 0.007, OR = 5.081), the presence of hypertension (*P* = 0.011, OR = 3.527), age (*P*<0.001, OR = 1.181), GA (*P* = 0.002, OR = 1.089) and diabetes duration (*P* = 0.008, OR = 1.074). The prevalence of coronary artery disease, cerebral infarction and LLI increased with cystatin C (*P*<0.01), and the prevalence of LLI in patients with cystatin C >1.2 mg/L was much higher than other three quartile groups. Receiver operating characteristic curve analysis revealed the cut point of cystatin C for LLI was 1.2 mg/L. The risk of LLI dramatically increased in patients with cystatin C >1.2 mg/L (OR = 21.793, 95% confidence interval 10.046−47.280, *P*<0.001). After adjusting for sex, age, duration, HbA1c, GA and hypertension, its OR still remained 3.395 (95% confidence interval 1.335–8.634).

**Conclusions:**

There was a strong and independent association between cystatin C and limb arterial disease in diabetic population, and cystatin C >1.2 mg/L indicated a great increased risk of LLI.

## Introduction

Lower limb ischemia (LLI) caused by peripheral arterial disease (PAD) is the major risk factor of foot ulceration and amputation which severely impairs the patients’ life quality. In the diabetic population, the onset of vascular abnormalities, atherosclerosis and plaque may be earlier and more obvious. [1] Therefore, it is vital for diabetic patients to recognize LLI and to control the risk factors of PAD as early as possible. It is well known that elder age, diabetes duration, cigarette smoking, poor glucose control, hypertension and hyperlipidemia are the traditional risk factors of arteriosclerosis for diabetic patients. [1] C-reactive protein (CRP), reflecting systemic inflammation, was one of the classical biomarkers for increasing risk of PAD. [2], [3] However, to our knowledge, the long-term predictive value of CRP for PAD was limited.

Cystatin C, an endogenous marker of renal function, is becoming to be more sensitive than creatinine in estimating glomerular filtration rate (GFR).[4]–[6] It is a cysteine-protease inhibitor that is produced by human nucleated cells, freely filtered at the glomerulus and reabsorbed in proximal renal tubular cells. [7] Apart from renal function, cystatin C recently has been found associated with cardiovascular events.[8]–[10] And cystatin C was proved to be predictor for incidence of PAD events in elder people in the community [11] and cardiovascular mortality in PAD population. [12] However, there is limited data about the association between serum cystatin C levels and LLI in type 2 diabetic patients. The purpose of this study is to explore the association between cystatin C and PAD, and to clarify its clinical value for LLI in Chinese patients who suffered from type 2 diabetes mellitus (T2DM).

## Subjects and Methods

### Ethics Statement

The study was approved by the Ethics Committee of Shanghai Jiaotong University Affiliated Sixth People’s Hospital and in accordance with the principle of the Helsinki Declaration. Written informed consent was obtained from all subjects.

### Subjects

A total of 1609 patients with type 2 diabetic mellitus (T2DM) were recruited from Shanghai Clinical Center of Diabetes at Shanghai Jiaotong University Affiliated Sixth People’s Hospital from July 2011 to June 2012. The diagnosis of diabetes mellitus was performed according to the 1999 World Health Organization criteria and 2012 American diabetes association standards. [13] The patients with type 1 diabetes, other specific types of diabetes and acute complications of diabetes mellitus were excluded. There were 914 males (56.8%) and 695 females (43.2%), with an average age of 60±12 years and diabetes duration of 9±7 years.

### Measurements and Information Collection

Clinical information, including sex, age, diabetes duration, height, weight, systolic blood pressure, diastolic blood pressure, previous history of smoking habit, hypertension (HTN), coronary artery disease (CAD), cerebral infarction (CI) and medication of aspirin or lipid lowering drugs was collected. Body mass index (BMI) was calculated as body weight (in kg) divided by the square of the height (in m). HTN was defined as systolic blood pressure ≥140 mmHg, diastolic blood pressure ≥90 mmHg or history of antihypertensive medicine administration. CAD was defined as a history of myocardial infarction or coronary arterial stenosis >50%. CI was defined as a history of ischemia attack showed by cerebral CT or MRI scan. Ankle-brachial index (ABI) was calculated by dividing the higher systolic blood pressure in the dorsalis pedis and posterior tibial artery by the higher of the brachial systolic blood pressure. The lower value of ABI in either limb was used for analysis. The arterial lesion of carotid and lower extremity artery were evaluated by color Doppler ultrasound examination, and the carotid and femoral intima-media thickness (IMT) were collected. LLI was defined by ABI <0.9 and lower extremity arterial stenosis >50%. The patients with ABI ≥0.9 and <1.0 or ABI >1.3 were excluded due to uncertainty status about blood supply. Glomerular filtration rate (GFR) was measured by the ^99m^Tc-DTPA renal dynamic imaging using Siemens Signature e.cam SPECT (General Electric Medical Systems, Waukesha, WI, USA).

Serum cystatin C concentration was determined by high sensitive latex-enhanced immune-turbidimetric method with an automatic biochemical analyzer (7600–020; Hitachi Inc., Tokyo, Japan). The distribution of serum cystatin C levels was at a median of 1.0 mg/L (inter-quartile range 0.3 mg/L). Patients were also subdivided into four groups by cystatin C quartile: ≤0.8 (n = 259), 0.8–1.0 (n = 502), 1.0–1.2 (n = 368) and >1.2 (n = 479).

White blood cell (WBC), red blood cell (RBC), hemoglobin (Hb), platelet (PLT) and neutrophils (N) were measured by an automated blood analyzer (XE-5000; Sysmex Corp., Japan). C-reactive protein was measured by Dad Behring Nephelometer II System (antiserum to human albumin; Siemens Healthcare Diagnostics). Fasting plasma glucose (FPG) and 2-h postprandial blood glucose (PPG) were measured by glucose oxidase method (Automatic Biochemistry Analyzer; Beckman Coulter, USA). Glycosylated hemoglobin (HbA1c) was estimated by high-pressure liquid chromatography using the Variant™ II machine (Bio-Rad Inc., Hercules, CA). Glycosylated serum albumin (GA) was determined by the liquid enzymatic assay using the Glamour 2000 automatic biochemical analyzer (MD Inc., Silicon Valley, California, USA). Total protein (TP), albumin (Alb), alanine aminotransferase (ALT), aspartate aminotransferase (AST), alkaline phosphatase (AKP), γ-glutamyl transpeptidase (γ-GT), blood urea nitrogen (BUN), serum creatinine (Cr), uric acid (UA) and serum lipids, including total cholesterol (TC), triglyceride (TG), high-density lipoprotein cholesterol (HDL-C), low-density lipoprotein cholesterol (LDL-C) were assessed by enzymatic method with an automatic biochemical analyzer (7600–020; Hitachi Inc., Tokyo, Japan).

### Statistical Analysis

All the statistical analysis was performed using SPSS 16.0 (SPSS Inc., Chicago, IL). Data were expressed as mean ± SD for continuous variables, and as percentages for categorical variables. Between LLI and without LLI, and among cystatin C quartiles, continuous variables were compared by the Mann-Whitney *U* test and categorical variables were compared by the χ^2^ test. Binary logistic regression analysis was used to find the independent risk factors for LLI. Receiver operating characteristic curve (ROC) was made to find the cut-off point of cystatin C for predicting LLI. Binary logistic regression analysis was used to calculate the ORs and 95% confidence intervals for LLI by cystatin C quartiles. *P*<0.05 was considered statistically significant.

## Results

### Comparison of Clinical Characteristics between Patients with and without Lower Limb Ischemia

The clinical characteristics between patients with and without LLI were showed in Table 1. There were significant differences in age, duration of diabetes, systolic blood pressure, GA, CRP, RBC, Hb, neutrophils, TP, albumin, BUN, Cr, cystatin C, GFR, TC, HDL-C, LDL-C, ALT, AKP, carotid and femoral IMT, prevalence of HTN, CAD and CI (all *P*<0.01) and HbA1c, AST, medication of aspirin and lipid lowering drugs (all *P*<0.05) between patients with and without LLI. There was no difference in BMI, FPG, PPG, PLT, WBC, UA, TG and γ-GT between two groups (all *P*>0.05). The serum concentrations of cystatin C were 1.53±0.60 and 1.08±0.30 mg/L in patients with and without LLI, respectively.

**Table 1:** Comparison of clinical characteristics between type 2 diabetic patients with and without lower limb ischemia.

	With LLI	Without LLI	*P*
n (male/female)	270 (147/123)	1251 (710/541)	/
Age (years)	73±8	57±11	<0.001
Duration (years)	14±8	8±6	<0.001
BMI (kg/m^2^)	24.67±3.30	25.17±3.64	0.104
Smoking (%)	26.6	32.6	0.038
HTN (%)	67.5	48.9	<0.001
CAD (%)	28.9	9.1	<0.001
CI (%)	30.9	6.9	<0.001
Aspirin (%)	65.2	53.9	0.026
Lipid lowering drugs (%)	41.7	53.1	0.026
SBP (mmHg)	138.11±19.19	132.60±17.74	0.001
DBP (mmHg)	78.85±10.06	81.12±10.06	0.020
FPG (mmol/L)	7.95±2.81	8.00±2.71	0.649
PPG (mmol/L)	12.71±4.27	12.92±4.31	0.742
HbA1c (%)	8.47±2.00	8.14±1.97	0.019
GA (%)	23.19±7.33	21.71±6.68	0.003
CRP (mg/L)	7.61±17.27	3.24±11.74	0.003
RBC (×10^9^)	4.35±0.52	4.65±0.53	<0.001
Hb (g/L)	130.47±18.10	140.48±16.22	<0.001
PLT (×10^9^)	194.01±54.24	198.97±52.31	0.331
WBC (×10^9^)	6.37±1.75	6.15±1.62	0.202
N (%)	64.69±8.58	61.19±8.79	<0.001
TP (g/L)	66.47±5.62	67.90±5.75	0.001
Alb (g/L)	41.92±4.39	44.44±3.63	<0.001
BUN (mmol/L)	6.39±2.73	5.27±1.49	<0.001
Cr (µmol/L)	83.25±42.33	65.99±18.73	<0.001
UA (µmol/L)	329.39±98.97	315.59±84.00	0.124
Cystatin C (mg/L)	1.53±0.60	1.08±0.30	<0.001
GFR (ml/min)	72.39±25.35	99.31±24.69	<0.001
TC (mmol/L)	4.59±1.15	4.87±1.11	<0.001
TG (mmol/L)	1.70±1.40	1.89±1.69	0.054
HDL-C (mmol/L)	1.04±0.28	1.10±0.28	0.001
LDL-C (mmol/L)	2.65±0.84	2.85±0.84	0.001
ALT (U/L)	18.88±11.44	25.21±17.15	<0.001
AST (U/L)	19.99±12.50	21.27±10.55	0.037
AKP (U/L)	73.32±25.81	65.94±19.38	<0.001
γ-GT (U/L)	32.64±27.59	34.62±31.99	0.348
Carotid IMT (mm)	0.96±0.25	0.82±0.18	<0.001
Femoral IMT (mm)	1.09±0.19	0.91±0.20	<0.001

LLI, lower limb ischemia; Duration, duration of diabetes; BMI, body mass index; HTN, hypertension; CAD, coronary artery disease; CI, cerebral infarction; SBP, systolic blood pressure; DBP, diastolic blood pressure; FPG, fasting plasma glucose; PPG, 2-h postprandial blood glucose; HbA1c, glycosylated hemoglobin; GA, glycosylated serum albumin; CRP, C-reactive protein; RBC, red blood cell; Hb, hemoglobin; PLT, platelet; WBC, white blood cell; N, neutrophils; TP, total protein; Alb, albumin; BUN, blood urea nitrogen; Cr, serum creatinine; UA, uric acid; GFR, glomerular filtration rate; TC, total cholesterol; TG, triglyceride; HDL-C, high-density lipoprotein cholesterol; LDL-C, low-density lipoprotein cholesterol; ALT, alanine aminotransferase; AST, aspartate aminotransferase; AKP, alkaline phosphatase; γ-GT, γ-glutamyl transpeptidase; IMT, intima-media thickness.

### The Risk Factors for Lower Limb Ischemia

Taking LLI as the dependent variable, the risk factors mentioned above were entered into binary logistic regression analysis. The results showed that the significant risk factors included cystatin C *(P* = 0.007, OR = 5.081), the presence of hypertension (*P* = 0.011, OR = 3.527), age (*P*<0.001, OR = 1.181), GA (*P* = 0.002, OR = 1.089) and diabetes duration (*P* = 0.008, OR = 1.074) (Table 2).

**Table 2:** Risk factors for LLI by binary logistic regression analysis.

	B	S.E.	Wald	*P*	OR	95% CI for OR	
						Lower	Upper
Cystatin C	1.626	0.602	7.290	0.007	5.081	1.561	16.536
HTN	1.260	0.495	6.472	0.011	3.527	1.336	9.313
Age	0.166	0.028	34.89	<0.001	1.181	1.118	1.248
GA	0.085	0.027	9.874	0.002	1.089	1.033	1.148
Duration	0.071	0.027	6.943	0.008	1.074	1.018	1.132

CI, confidence interval; HTN, hypertension; GA, glycosylated serum albumin; Duration, duration of diabetes.

### Comparison of Characteristics by Cystatin C Quartiles

**Figure 1:**
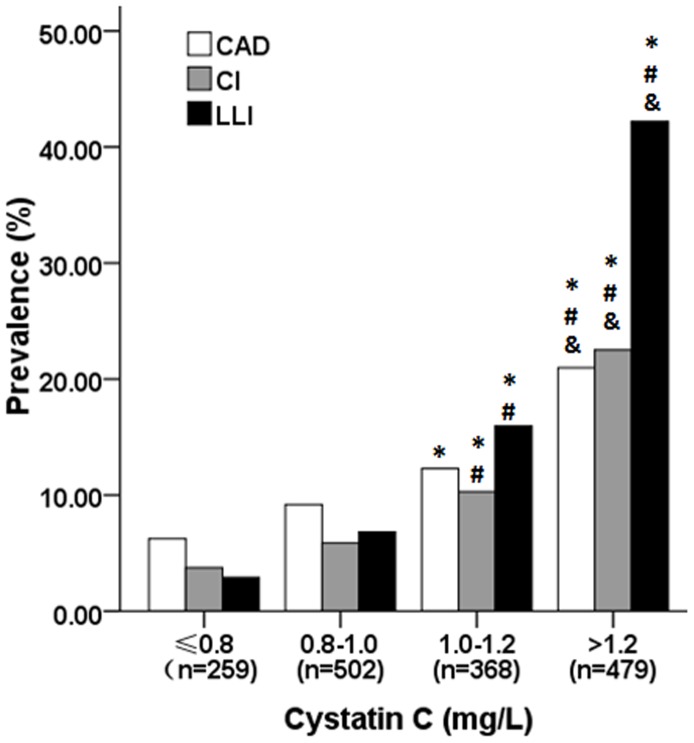
Prevalence of coronary artery disease, cerebral infarction and lower limb ischemia by cystatin C quartiles. *P<0.01, compared with the first quartile; ^#^ *P*<0.01, compared with the second quartile; ^&^ *P*<0.01, compared with the third quartile. CAD, coronary artery disease; CI, cerebral infarction; LLI, lower limb ischemia.

As the concentration of cystatin C rose, the levels of age, diabetes duration, carotid and femoral IMT, systolic blood pressure, neutrophils, BUN, Cr, UA and CRP increased, but RBC, Hb, PLT, albumin, FPG, PPG and GFR decreased (Table 3). The proportions of CAD, CI and LLI ascended with cystatin C, and the prevalence of LLI in patients in the highest cystatin C quartile aggravated dramatically compared to other three quartile groups (Figure 1).

**Table 3:** Comparison of clinical characteristics among four cystatin C groups.

Cystatin C	≤0.8	0.8–1.0	1.0–1.2	>1.2	*P*
n (M/F)	259(104/155)	502(295/207)	368(222/146)	479(292/187)	/
Age (yrs)	52±10	55±11	61±11	68±10	<0.001
Duration (yrs)	7±5	8±6	10±7	12±8	<0.001
BMI (kg/m^2^)	24.81±3.75	25.14±3.66	25.25±3.52	25.10±3.45	0.347
Smoking (%)	24.2	33.9	32.5	30.6	0.042
Hypertension (%)	38.7	47.3	52.5	67.2	<0.001
SBP (mmHg)	129.61±16.33	130.18±14.93	133.93±18.89	138.48±20.52	<0.001
DBP (mmHg)	81.40±10.19	80.68±9.10	79.89±958	81.29±11.53	0.343
FPG (mmol/L)	9.15±3.14	8.11±2.81	7.78±2.48	7.47±2.46	<0.001
PPG (mmol/L)	14.09±4.38	13.14±4.51	12.19±3.84	12.51±4.31	<0.001
HbA1c (%)	8.78±2.22	8.06±1.91	7.74±1.84	8.36±1.94	<0.001
GA (%)	23.77±7.37	21.52±6.77	20.64±6.18	22.35±6.71	<0.001
CRP (mg/L)	3.28±16.18	3.05±9.71	3.78±11.31	5.59±14.71	0.004
RBC (×10^9^)	4.68±0.44	4.72±0.49	4.60±0.50	4.42±0.64	<0.001
Hb (g/L)	140.05±15.30	143.10±15.55	139.03±15.29	132.3±19.11	<0.001
PLT (×10^9^)	214.13±53.13	200.01±53.84	198.11±49.72	187.72±51.21	<0.001
WBC (×10^9^)	6.15±1.91	6.26±1.50	6.18±1.72	6.16±1.59	0.279
N (%)	60.96±8.66	60.99±8.88	61.24±9.05	63.28±8.59	0.009
TP (g/L)	68.18±5.61	68.64±5.62	67.80±5.57	66.50±5.97	<0.001
Alb (g/L)	44.92±3.49	45.00±3.30	44.24±3.50	42.26±4.48	<0.001
BUN (mmol/L)	4.79±1.19	5.04±1.18	5.29±1.33	6.60±2.57	<0.001
Cr (µmol/L)	53.12±11.96	61.07±12.30	68.68±14.25	88.77±36.56	<0.001
UA (µmol/L)	277.40±71.21	304.60±79.50	324.98±78.30	353.05±97.73	<0.001
Cystatin C (mg/L)	0.75±0.07	0.95±0.05	1.14±0.05	1.62±0.47	<0.001
GFR (ml/min)	114.96±18.48	105.59±21.01	93.75±22.31	71.81±22.99	<0.001
TC (mmol/L)	5.04±1.02	4.84±1.08	4.71±1.13	4.75±1.20	<0.001
TG (mmol/L)	1.98±2.30	1.79±1.47	1.85±1.51	1.82±1.32	0.860
HDL-C (mmol/L)	1.17±0.30	1.11±0.27	1.06±0.27	1.05±0.29	<0.001
LDL-C (mmol/L)	2.99±0.85	2.85±0.81	2.73±0.84	2.75±0.89	0.001
ALT (U/L)	24.59±16.14	26.51±17.75	24.23±17.06	21.00±13.84	<0.001
AST (U/L)	19.95±12.01	21.66±9.96	21.43±9.53	20.77±11.74	0.001
AKP (U/L)	65.19±20.42	67.30±18.55	66.39±19.57	69.97±24.15	0.208
γ-GT (U/L)	33.38±37.85	36.48±31.55	32.44±26.84	34.13±30.09	0.116
Carotid IMT (mm)	0.78±0.17	0.81±0.21	0.85±0.19	0.89±0.20	<0.001
Femoral IMT (mm)	0.87±0.25	0.88±0.21	0.95±0.17	1.02±0.19	<0.001

Duration, duration of diabetes; BMI, body mass index; SBP, systolic blood pressure; DBP, diastolic blood pressure; FPG, fasting plasma glucose; PPG, 2-h postprandial blood glucose; HbA1c, glycosylated hemoglobin; GA, glycosylated serum albumin; CRP, C-reactive protein; RBC, red blood cell; Hb, hemoglobin; PLT, platelet; WBC, white blood cell; N, neutrophils; TP, total protein; Alb, albumin; BUN, blood urea nitrogen; Cr, serum creatinine; UA, uric acid; GFR, glomerular filtration rate; TC, total cholesterol; TG, triglyceride; HDL-C, high-density lipoprotein cholesterol; LDL-C, low-density lipoprotein cholesterol; ALT, alanine aminotransferase; AST, aspartate aminotransferase; AKP, alkaline phosphatase; γ-GT, γ-glutamyl transpeptidase; IMT, intima-media thickness.

### The Diagnostic Value of Cystatin C for Lower Limb Ischemia

**Figure 2:**
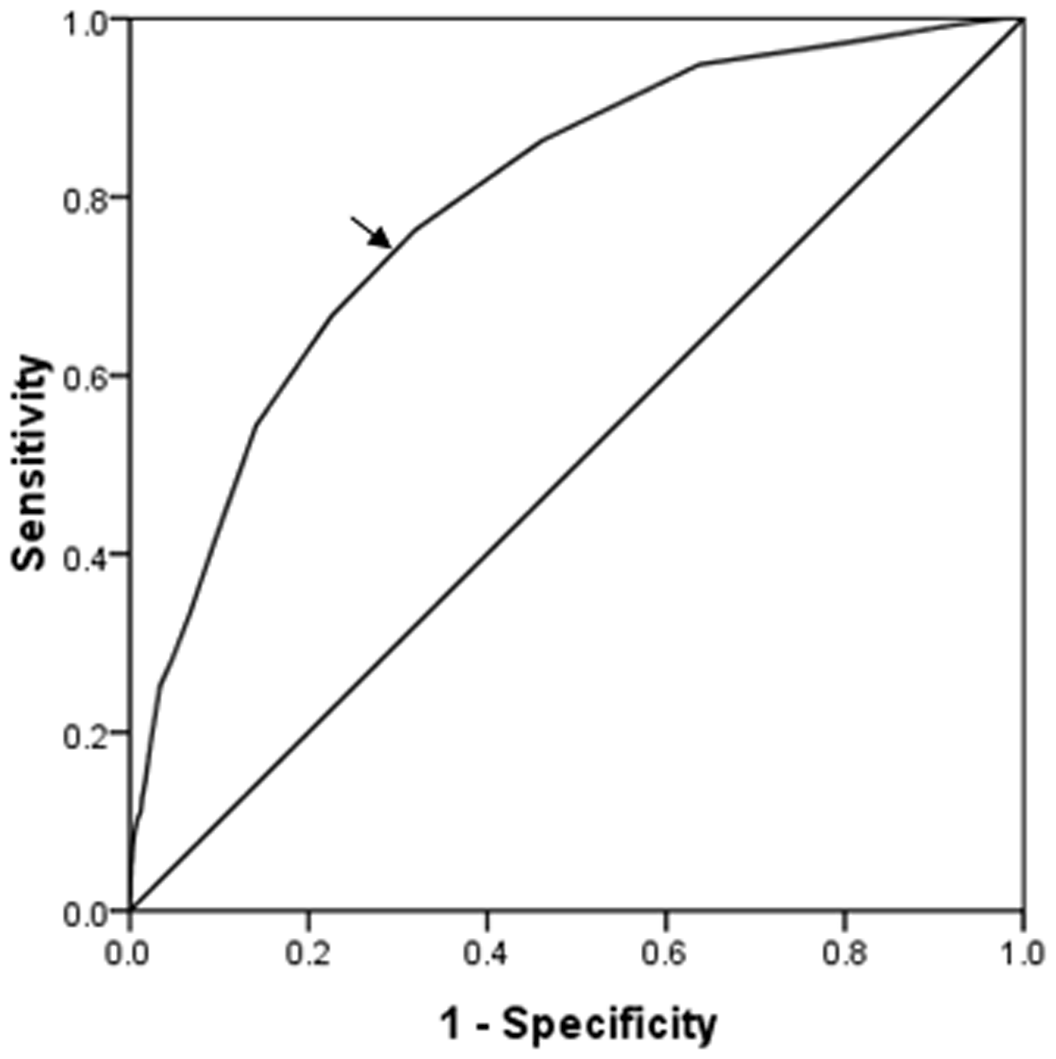
Receiver operating characteristic curve analysis of cystatin C cut point for the presence of lower limb ischemia. Area under curve = 0.794 (*P*<0.001), 95% confidence interval: 0.765–0.823; Identified cystatin C cut point = 1.2 mg/L, Youden index = 0.44; Sensitivity: 76%; Specificity: 68%.

ROC curve analysis was performed to verify the diagnostic accuracy of cystatin C for LLI. The area under curve (AUC) was 0.794 (95% confidence interval 0.765–0.823); the optimal cut point of cystatin C was 1.2 mg/L (Figure 2). And sensitivity was 76%; specificity was 68% at this level. The ORs and 95% confidence intervals for LLI by cystatin C quartiles were shown in Table 4. OR for LLI increased 20-fold in patients in the highest cystatin C level than that in the lowest quartile group (OR = 21.793, 95% confidence interval 10.046−47.280, *P*<0.001). After adjusting for sex, age, duration, HbA1c, GA and hypertension, its OR was 3.395 (95% confidence interval 1.335–8.634, *P*<0.001).

**Table 4:** Odds Ratios and 95% confidence intervals of lower limb ischemia by cystatin C quartiles.

Cystatin C (mg/L)	OR (95% CI)	*P*	Adjusted OR (95% CI)*	*P*
≤0.8	1 (reference)	/	1 (reference)	/
0.8–1.0	2.355 (1.019−5.442)	0.045	1.271 (0.478–3.379)	0.631
1.0–1.2	6.155 (2.748−13.787)	<0.001	1.792 (0.683–4.702	0.236
>1.2	21.793 (10.046−47.280)	<0.001	3.395 (1.335–8.634)	0.010

CI, confidence interval.

* Adjusting for sex, age, diabetes duration, hypertension, HbA1c and GA.

## Discussion

This cross-sectional study including 1609 Chinese type 2 diabetic patients at the first time investigated the relationship between serum cystatin C levels and lower extremity arterial disease in T2DM population. It was revealed that cystatin C was strongly and independently associated with the prevalence of diabetic lower arterial lesion. Moreover, cystatin C higher than 1.2 mg/L indicated a dramatically increased risk of LLI. Therefore, cystatin C was believed to be a sensitive biomarker for screening out PAD in the type 2 diabetic populations.

LLI is one of the manifestations of PAD in diabetes, caused by arteriosclerosis, plaque formation and arterial occlusion. As we all know, in diabetic population, the traditional risk factors for PAD are smoking, elder age, advanced duration of diabetes, poor blood glucose control, hypertension and hyperlipidemia. [1] Similarly, in this study, we found that there were significant differences in age, duration of diabetes, blood glucose indicators, the prevalence of HTN, CAD and CI between patients with and without LLI. However, the indexes reflecting blood lipid and hepatic function were in lower level in patients with LLI. It may be due to the diversity of patients’ selection and the poor nutrition state in patients with LLI. Besides the traditional risk factors we also found that patients with lower limb arterial lesion had higher level of AKP and neutrophils, but lower concentrations of hemoglobin and serum albumin, which was consistent with previous studies.[14]–[17].

We also revealed that renal function indicators in those with LLI were much higher than those without renal dysfunction and the degree of peripheral arterial lesion was significantly correlated with renal function and GFR. Several previous studies reported a close association between chronic renal insufficiency and PAD.[18]–[20] In a cross-sectional study of 1461 Taiwanese type 2 diabetic outpatients, a low estimated GFR was found to be a major risk factor for low ABI. [21] Another cross-sectional study in general population in the National Health and Nutrition Examination Survey (NHNES) 1999 to 2002 also demonstrated that GFR estimated using cystatin C was strongly associated with PAD. [22] The possible mechanisms underlying the close relationship between renal disease and PAD were the co-progression of renal artery stenosis and overall atherosclerosis including lower extremity vascular disease, [23] and excessive accumulation of metabolic wastes due to reduced GFR. [24].

Cystatin C is a small-molecular-weight non-glycosylated basic protein produced steadily by human nucleated cells. Because cystatin C is freely filtered at the glomerulus at a constant rate and is less affected by sex, age and diet, [25] it is becoming an ideal endogenous marker of GFR.[4]–[6] Recently, cystatin C has been reported to be a predictor for future cardiovascular events including coronary heart disease, lower extremity arterial disease and mortality, independent of renal function. [26], [27] In a community-based, prospective study in elderly adults, the subjects with cystatin C higher than 1.27 mg/L had a 1.9-fold increased new onset of PAD events after semi-annually follow up. [11] However, there was no research to explore the association between cystatin C and the peripheral artery diseases of lower limbs in diabetic population so far. In the present study, cystatin C was one of the independent risk factors for LLI. The optimal cut point of cystatin C for LLI was 1.2 mg/L, and the sensitivity was 76% and the specificity was 68%. Cystatin C higher than 1.2 mg/L reflected a 3-fold increased risk of LLI after adjusting for sex, age, diabetes duration, HbA1c, GA and hypertension. This optimal cut point was close to that in the study above, but the odds ratio at this level was much higher. Therefore, it was believed that cystatin C was notably associated with PAD in Chinese diabetic patients.

Although the mechanism linking cystatin C with cardiovascular disease was currently not well known, numerous studies tried to explain it. Urbonaviciene et al. observed a significant correlation of cystatin C and high-sensitivity C-reactive protein (hs-CRP) suggesting chronic low-grade inflammation was one of mechanism concerned, [12] consistent with several previous studies. [11], [26], [28] In the present study, CRP levels were also found much higher in patients with LLI, but it was not been shown an independent risk factor in logistic regression analysis. In a study of 478 type 2 diabetic patients, insulin resistance and biomarkers reflecting inflammation, including urine albumin-creatinine ratio, uric acid and homocysteine, were implicated in the link between cystatin C and coronary heart disease. [29] However, another case-control study found that cystatin C remained higher in PAD patients even corrected for eGFR, CRP and IL-6. [30] In several earlier studies, decreased expression of cystatin C was also found in atherosclerotic lesions, and lower cystatin C levels in cerebral infarction patients. [31], [32] It was possible that cystatin C, a cathepsin inhibitor, increased to counteract the vascular impairment of cathepsin. [7].

Diabetic lower extremity arterial disease was one of the major causes for foot ulceration and amputation. Early detection and treatment of lower extremity disease is critical to prevent amputation and mortality for diabetic population. From the results of the present study, cystatin C levels were strongly associated with PAD. The prevalence of CAD, CI and LLI increased with cystatin C quartiles, especially the prevalence of LLI, suggesting that cystain C was more suitable to be a PAD predictor. From the aforementioned results, cystatin C was not only an ideal marker reflecting early phase impairment of renal function, but also has great potential advantages to predict the progression of arteriosclerosis in diabetic population. Although the present study couldn’t provide explicit answer to the detailed mechanism linking cystatin C and peripheral arterial disease, further follow-up and basic research will be carried out to reveal its related mechanism. The exploration of the possible mechanisms underlying cystatin C and cardiovascular disease may provide more approaches to prevent and treat macrovascular disease in the future.

The strength of this study is that GFR was directly measured by the ^99m^Tc-DTPA renal dynamic imaging rather than estimated from serum creatinine, which was more accurate and avoided the impact from serum creatinine. One of the limitations was the relatively small sample size, especially the patients with critical limb ischemia, thus the patients could not be further divided to several subgroups based on low ABI values. Another limitation was that it was a cross-sectional research lacking the outcome of lower extremities in patients with high cystatin C. Therefore, further follow-up data is needed to clarify the predictive value of cystatin C on the LLI and foot diseases.

In conclusion, apart from renal function, serum cystatin C levels had a very close connection with PAD in Chinese T2DM patients. The optimal cutpoint of cystatin C was higher than 1.2 mg/L for reflecting more than 20 times increased risk of LLI. The detection of cystatin C concentration is of great value for screening out the patients with the angiostenosis risk of lower limb to prevent foot ulceration and amputation. The discovery of the detailed mechanisms underlying cystatin C and vascular abnormalities may be helpful to explore the novel therapeutic strategy and evaluation of curative effect for diabetic LLI and other macrovascular complications.
